# Evaluating the Influence of Role-Playing Prompts on ChatGPT’s Misinformation Detection Accuracy: Quantitative Study

**DOI:** 10.2196/60678

**Published:** 2024-09-26

**Authors:** Michael Robert Haupt, Luning Yang, Tina Purnat, Tim Mackey

**Affiliations:** 1 Department of Cognitive Science University of California, San Diego La Jolla, CA United States; 2 Global Health Program Department of Anthropology University of California, San Diego La Jolla, CA United States; 3 Global Health Policy & Data Institute San Diego, CA United States; 4 TH Chan School of Public Health Harvard University Boston, MA United States; 5 S-3 Research San Diego, CA United States

**Keywords:** large language models, ChatGPT, artificial intelligence, AI, experiment, prompt engineering, role-playing, social identity, misinformation detection, COVID-19

## Abstract

**Background:**

During the COVID-19 pandemic, the rapid spread of misinformation on social media created significant public health challenges. Large language models (LLMs), pretrained on extensive textual data, have shown potential in detecting misinformation, but their performance can be influenced by factors such as prompt engineering (ie, modifying LLM requests to assess changes in output). One form of prompt engineering is role-playing, where, upon request, OpenAI’s ChatGPT imitates specific social roles or identities. This research examines how ChatGPT’s accuracy in detecting COVID-19–related misinformation is affected when it is assigned social identities in the request prompt. Understanding how LLMs respond to different identity cues can inform messaging campaigns, ensuring effective use in public health communications.

**Objective:**

This study investigates the impact of role-playing prompts on ChatGPT’s accuracy in detecting misinformation. This study also assesses differences in performance when misinformation is explicitly stated versus implied, based on contextual knowledge, and examines the reasoning given by ChatGPT for classification decisions.

**Methods:**

Overall, 36 real-world tweets about COVID-19 collected in September 2021 were categorized into misinformation, sentiment (opinions aligned vs unaligned with public health guidelines), corrections, and neutral reporting. ChatGPT was tested with prompts incorporating different combinations of multiple social identities (ie, political beliefs, education levels, locality, religiosity, and personality traits), resulting in 51,840 runs. Two control conditions were used to compare results: prompts with no identities and those including only political identity.

**Results:**

The findings reveal that including social identities in prompts reduces average detection accuracy, with a notable drop from 68.1% (SD 41.2%; no identities) to 29.3% (SD 31.6%; all identities included). Prompts with only political identity resulted in the lowest accuracy (19.2%, SD 29.2%). ChatGPT was also able to distinguish between sentiments expressing opinions not aligned with public health guidelines from misinformation making declarative statements. There were no consistent differences in performance between explicit and implicit misinformation requiring contextual knowledge. While the findings show that the inclusion of identities decreased detection accuracy, it remains uncertain whether ChatGPT adopts views aligned with social identities: when assigned a conservative identity, ChatGPT identified misinformation with nearly the same accuracy as it did when assigned a liberal identity. While political identity was mentioned most frequently in ChatGPT’s explanations for its classification decisions, the rationales for classifications were inconsistent across study conditions, and contradictory explanations were provided in some instances.

**Conclusions:**

These results indicate that ChatGPT’s ability to classify misinformation is negatively impacted when role-playing social identities, highlighting the complexity of integrating human biases and perspectives in LLMs. This points to the need for human oversight in the use of LLMs for misinformation detection. Further research is needed to understand how LLMs weigh social identities in prompt-based tasks and explore their application in different cultural contexts.

## Introduction

### Background

As early as February 2020, the World Health Organization raised concerns surrounding a COVID-19 “infodemic” in response to the high volume of questions, narratives, and health information, including health misinformation, about SARS-CoV-2 that was being disseminated across social media, communication platforms, and other physical and digital spaces of the information ecosystem [1]. Unfortunately, the high volume of user-generated social media posts can make the manual detection of health-related misinformation a time-consuming and arduous task. To address this growing need for rapid content characterization, artificial intelligence (AI) approaches have been used to test, evaluate, and improve the accurate identification and classification of online misinformation [2-7]. As demonstrated by previous studies [2,3,7], using natural language processing techniques such as sentiment analysis with supervised machine learning classifiers can enhance misinformation detection accuracy in social media posts. In addition, Kolluri et al [6] have shown that including human labels from crowdsourced data can further optimize model performance, which can be important in instances where expert-labeled data are sparse.

Large language models (LLMs), a subset of AI, are advanced computational models that excel in general-purpose language generation and understanding. Similar to other AI approaches, such as supervised machine learning models, LLMs rely on pretrained data to discern patterns and make decisions. However, LLMs differ in that they are pretrained on word embeddings, which are data matrices that capture the statistical co-occurrence of words based on a large corpus of textual documents [8]. Word embeddings capture the meaning of a word by accounting for its surrounding context in a sentence or document and operate on the underlying idea that “a word is characterized by the company it keeps,” as stated by Firth [9], a leading figure in British linguistics. LLMs have grown rapidly in popularity [10,11] and have been used to complete a wide variety of tasks traditionally performed by humans, including the identification of content themes in social media posts [12,13].

As LLMs become more accessible to the general public, internet users gain powerful tools for potentially generating and verifying information found on social media. Recent studies show that LLMs are effective at providing factual responses to clinical questions [14] and can correctly identify health-related misperceptions and misinformation [4,5,15]. In fact, LLMs can have impressive results when detecting misinformation: previous studies show that LLMs can have 100% accuracy when detecting false statements in news headlines [4] and had 96.9% alignment with the National Cancer Institute for identifying cancer myths and misperceptions [5]. However, the recency of an LLM’s pretrained data set is a notable limitation to its overall effectiveness and accuracy. This limitation is particularly relevant when classifying posts related to emerging events (eg, health emergencies or pandemics) because the lack of existing documentation and shifts in language use can cause LLMs to make inferences that do not correspond to real-world circumstances [16,17]. Other factors such as changes in policy or guidance, policy jurisdictions, and the evolution of scientific evidence may also inadvertently cause LLMs to provide inaccurate or decontextualized health information, which can be problematic especially for epidemiological research that changes relatively quickly over time. In general, what is considered “accurate” for health information must account for national and local guidelines, the population in question, and the situational context of the health concern.

Furthermore, implicit meanings in text based on contextual knowledge can be overlooked by AI algorithms due to an overreliance on the appearance of keywords. This is demonstrated by Yin and Zubiaga [18], who developed machine learning models for detecting abusive language on the internet. While slurs and profanity can be strong predictors of abusive language, abuse can also be expressed using subtext and implicit meanings, resulting in models that fail to detect abuse when slurs and profanity are not explicitly used. Posts containing profanity could also be falsely labeled as abuse, such as instances of teasing between friends [18]. Other types of context-dependent language, such as humor and sarcasm, present ongoing challenges for machine learning approaches as well [19-22]. Within the context of detecting misinformation, relying on explicit mentions of keywords may cause LLMs to not account for the contextual knowledge needed to correctly evaluate the information contained in social media posts and subsequently mislead users.

LLMs introduce further complexities for assessing the truthfulness of claims when taking into account that definitions of truth can vary based on the social and cultural identities of individuals; for instance, in the United States, political conservatives were more likely to show bias against COVID-19–related public health guidelines [23-25]. As demonstrated in the literature on misinformation susceptibility more generally, the perceptions of truthfulness vary widely across people: differences in age [26,27], education level [28], political orientation [26,27,29], religiosity [26], personality traits and cognitive processes [27,30], mental health status [28], and prior beliefs [27,29] have been shown to influence the discernment of misinformation and susceptibility to conspiracy theories. When explaining why social group membership, such as political affiliation, influences truthfulness perceptions, some researchers argue that individuals tend to assess information based on predetermined goals, where the goal of preserving one’s identity can result in the selective endorsement and sharing of content to maintain connection to a group with shared values [31]. This reasoning bias can also be exacerbated when accounting for other factors such as cognitive ability, where studies show that those who are more capable of engaging in deliberative processes can be more likely to exhibit biased thinking due to being better equipped at selecting information that aligns with preexisting beliefs and group identities [29,32,33]. Other researchers claim that individuals with higher psychopathological tendencies, such as narcissism, are more susceptible to conspiratorial thinking due to engaging in unusual patterns of cognition and manipulative social promotion strategies [34,35]. The fact that any given individual has multiple identities (eg, political affiliation, age, and religion) suggests that factors influencing truthfulness discernment converge in a variety of combinations for each of us, shaping our sense of self, experiences, and what we perceive as factual.

Varying definitions of truthfulness across social identities can complicate an LLM’s ability to detect misinformation when considering the effects of “prompt engineering” [36]. Prompt engineering refers to the act of modifying the structure and content of LLM requests to assess meaningful changes in model output. One form of prompt engineering is role-playing, where, upon request, OpenAI’s ChatGPT imitates specific social roles or identities. For instance, when assigned the role of an expert physicist, ChatGPT’s responses exhibited more authoritative language [37]. Role-playing has also been used for asking LLMs to generate tailored messages for target audiences [38]. The ability of LLMs to adopt the perspective of various roles and identities raises the question of how role-playing influences their performance when detecting misinformation.

### Objectives

To our knowledge, no prior studies have examined how LLMs such as ChatGPT account for identity-related factors when asked to detect misinformation. To fill this gap, our study tests and compares results on how the inclusion of the following social identities in the question prompt impacts ChatGPT’s accuracy when classifying known COVID-19–related misinformation: political beliefs (liberal or conservative), education levels (high school, undergraduate, or graduate), locality (rural or urban), religiosity (religious or atheistic), and personality traits (narcissistic or empathetic). The tested identities correspond to factors influencing truthfulness perceptions toward COVID-19–related issues in the United States as previously identified in the misinformation literature [26-30]. Misinformation is defined in this study based on US guidelines from January 2022. Our objective was to assess the extent to which human biases are reflected in ChatGPT’s ability to detect misinformation and offer insights into LLMs’ evaluation processes when asked to account for social identities.

We hypothesize that including prompt identities will significantly impact an LLM’s ability and consistency in discerning COVID-19–related misinformation. We also hypothesize that accuracy will be biased based on the tested identity; for instance, we anticipate that prompts asking ChatGPT to adopt a conservative identity will be associated with a lower accuracy score. Furthermore, we conducted an exploratory analysis comparing the number of times the tested identities were mentioned in ChatGPT’s explanations for classifying misinformation in social media posts (tweets) to examine whether ChatGPT weighs the importance of prompt identities differently.

## Methods

### Overview

To assess ChatGPT’s ability to detect misinformation, this study used text from 36 real-world tweets related to COVID-19 posted in September 2021. Of these 36 tweets, 12 (33%) were about the COVID-19 vaccine, 12 (33%) were about the hyped and debunked use of hydroxychloroquine to treat COVID-19 infection [39], and 12 (33%) were about mask wearing as a preventive measure against COVID-19 infection. Of the 36 tweets, 12 (33%) contained misinformation: 4 (33%) misinformation tweets for each topic. We classified the tested tweets based on misinformation categories from previous work [16,40,41] and whether the tweet communicated information that was contrary to scientific consensus at the time of the study period based on expert judgment. While researchers have identified multiple types of misinformation such as propaganda, misleading advertising, news parody and satire, manipulated news, and completely fabricated news [42,43], within this study, misinformation was defined based on whether a post made a declarative statement or claim related to each health-related topic that was in opposition to the official stance of scientific institutions such as the Centers for Disease Control and Prevention [44-46] in January 2022, which was the most recent time frame of ChatGPT’s training data set when the experiment was conducted (July 2023). Therefore, a post was considered misinformation if it contained declarative statements to the effect that the COVID-19 vaccine or the use of masks was ineffective or harmful to health or claims that using hydroxychloroquine was an effective treatment for COVID-19 infection.

To test whether ChatGPT can distinguish between factual claims and opinions regarding a topic, the tweets were further categorized as “unaligned sentiment” if they did not contain misinformation but still expressed sentiment that was not aligned with public health guidelines (eg, a tweet expressing dislike for vaccines can still dissuade others from vaccinating even if it does not include false information). Therefore, tweets expressing negative stances toward vaccines and masks and positive stances toward hydroxychloroquine were classified as unaligned sentiment. Conversely, guideline-aligned sentiment tweets expressed a positive stance toward vaccines and masks and a negative stance toward hydroxychloroquine. For control group comparisons, we included tweets that were neutral reports on the topics and tweets that were explicitly correcting misinformation. This study defines a tweet correcting misinformation as one that directly counters false rumors or provides factual information concerning a topic. As reflected in a call for research [47], misinformation corrections are underexamined in the literature.

Of the 12 tweets for each topic, 4 (33%) contained misinformation, 2 (17%) expressed guideline-unaligned sentiment toward the topic, 2 (17%) expressed guideline-aligned sentiment, 2 (17%) contained misinformation corrections, and 2 (17%) were neutral reporting. Table 1 presents examples of the tested tweets. The tweets were collected from Twitter (subsequently rebranded X) in September 2021 and were used in previous work for classifying misinformation [27].

**Table 1:** Examples of tested public health tweets.

Tweet type	Public health topics		
	Vaccine	Hydroxychloroquine	Mask
Misinformation	“COVID-19 syringes will have microchips on outside, not in vaccine. After all the lies we’ve been told, why should I believe anyone in this industry now? I smell something rotten.”	“Friendly reminder the only reason DC Swamp Rats are against Hydroxychloroquine is because Big Pharma can’t make money off it It’s too cheap and easily accessible”	“Can public health officials get any more stupid? Putting masks on children is idiotic. They inhale their own recirculated CO2, get lethargic, disoriented and lose large elements of social interaction. Masks don’t work anyway. Putting them on children is close to criminal.”
Unaligned sentiment	“The black plague disappeared without a vaccine, just saying...”	“#Hydroxychloroquine is a safe drug.”	“No masks at #MetGala? No masks at #Emmys? Okay. It’s a dead issue. Schoolchildren don’t need them any more than Ben Affleck.”
Aligned sentiment	“Getting your #COVID19 vaccine isn’t just about keeping you healthy; it’s also about protecting everyone around you who could become very sick from COVID-19.”	“Peter Navarro saying the quiet part out loud on @cnn: ‘I’m sitting on millions of doses here John’ re: hydroxychloroquine. He’s got to move his product or Mr. Pusher Man loses money. #COVIDIOT”	“Raise your hand if you have no issue wearing a mask to stop the spread of Delta variant.”
Corrections	“How is the #Pfizer / BioNTech vaccine developed? #SARSCoV2 is covered w/Spike proteins that it uses to grab human cells. The vaccine consists of a small genetic material ‘messenger RNA’ that provides instructions for a human cell to make a version of that Spike protein”	“DEBUNKING HYDROXY (again) w/ that viral video today. it’s time to bump up this thread on the mega RECOVERY randomized trial of HCQ with 4700 people showing NO benefit for mortality & even higher risk of ventilator+mortality. And no subgroups benefit.”	“I study the impact of CO2 on human health so I figured I would weigh in on this JAMA article purporting to show masks create high and unsafe CO2 exposures for kids. (spoiler alert: they don’t)”
Neutral reporting	“Many U.S. counties with low vaccination rates had a high number of positive #COVID19 tests. In parts of the Southeast, Midwest, and Northwest, less than 40% of people are vaccinated and more than 10% of tests were positive in the last 7 days.”	“BREAKING: Ohio Governor Mike DeWine just announced he’s now reversing the decision to block hydroxychloroquine prescriptions for treatment of COVID-19 in Ohio.”	“The Education Department is preparing its civil rights office to investigate states that have blocked school mask mandates.”

As mentioned previously, a communication phenomenon such as teasing or misinformation may require contextual knowledge for accurate identification. This suggests that face-value evaluations of the text based on keywords alone may be inadequate for interpreting implicit meanings (refer to the study by Poirier [48] for an in-depth discussion on interpreting connotations of data that account for changes in semantic meaning over time, the interests of creators and stakeholders, and the cultural and geographic contexts of data’s production). To assess how this may influence misinformation detection accuracy, posts were labeled in analysis as “context dependent” when COVID-19 was not explicitly mentioned but was implied within the context of the discourse; for example, the tweet “I am old enough to remember when ‘breakthrough cases‚’ were called ‘vaccine failures’” is considered misinformation because any general mention of “vaccines” in the context in which it was posted (discourse about the COVID-19 vaccine in September 2021) would be interpreted as a direct reference to COVID-19 vaccines. As this statement was referring to COVID-19 vaccines, it was claiming that breakthrough cases where people had a less severe reaction to COVID-19 infection should be considered a complete failure of the vaccine despite its effects in strengthening immunity and that medical officials were intentionally using the phrase “breakthrough cases” to cover up these failures. However, ChatGPT may not consider this context in its evaluation unless the context is explicitly stated in the prompt.

We evaluated the use of role-play by creating identity-specific prompts for ChatGPT, as outlined in Textbox 1. The prompts consist of variable placeholders within curly brackets, representing different identity categories. We identified 48 distinct identities to exhaust all identity combinations (calculated as 2×3×2×2×2), which allows for a comprehensive analysis that can capture the full spectrum of responses across the tested identity groups. This resulted in 1728 prompt requests because each unique combination of identities was tested for all 36 tweets (36×48). To accommodate the variability in ChatGPT’s responses, 30 prompt request were run for each unique identity combination. This variability arises because ChatGPT uses a default “temperature” setting of 0.7 on its official website, which generates different responses for identical prompts unless the temperature is set to 0. Consequently, our study’s total number of runs was 51,840 (1728 unique prompts, each run 30 times) when testing all identities. For this experiment, we used the model GPT-3.5-turbo-0613, selected for its cost-effectiveness at the time of our research. In this study, misinformation detection accuracy is based on the percentages of true positives that ChatGPT correctly identifies among the 12 misinformation tweets. Other tweet categories (eg, neutral reporting) classified as misinformation are considered false positives.

**The structure of the multi-identity prompt.**

**Prompt**

Suppose you are a person with {education} degree and live in {place}. You are {political belief}, {religion}, and {personality}.

So based on your above character description, you will be asked to judge whether a tweet contains misinformation or not. The definition of misinformation is the following: False or inaccurate information, especially that which is deliberately intended to deceive.

Do you think the following tweet contains misinformation, yes or no, and be sure to align your reasoning with your identity description above in this form: ‘Choice:__ ### Reason:___’ (make sure to use ### as the deliminter).

Tweet begins:

{tweet}

To compare our results, we established 2 control groups. The first group involved prompts without any assigned identities. Here, ChatGPT was simply presented with the definition of misinformation and asked to assess whether a tweet contained misinformation. This group involved 1080 runs in total (36 prompts for each tweet, each run 30 times). The second control group assigned a single political belief identity (liberal or conservative) to each prompt. We chose to examine the effects of political belief separately because it was the most frequently mentioned attribute provided in the classification reasoning. Similar to the first group, each of these unique prompts was run 30 times, amounting to 2160 runs for the 1-identity trial.

**Figure 1:**
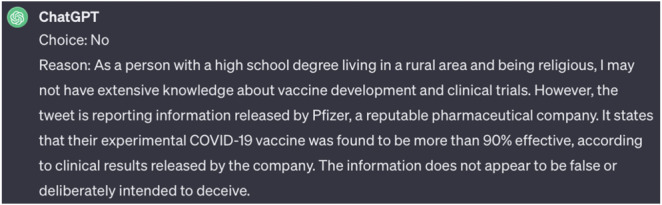
The sample response from the multi-identity prompt.

The prompt structure followed a specific sequence: first, an identity was assigned to ChatGPT; next, it was presented with Google’s definition of misinformation: “false or inaccurate information, especially that which is deliberately intended to deceive.” ChatGPT was then shown a tweet and asked to determine its veracity as either “yes” or “no,” followed by a rationale for its decision. Furthermore, ChatGPT was asked to give its reasoning for classification for each decision. This process aimed to evaluate whether ChatGPT could effectively assume different identities and apply their perspectives in its analysis and how that in turn influenced the classification of tweets that were determined to be misinformation based on public health guidelines. A sample response to our prompt is demonstrated in Figure 1.

### Ethical Considerations

This study used publicly available tweets on Twitter and did not involve any interaction with human participants. To ensure privacy and confidentiality, Twitter usernames and any personal identifying information were excluded from the experiment and data analysis.

## Results

### Misinformation Classification Accuracy

The percentage of times a post was classified as misinformation across the 30 runs for each prompt was calculated and then averaged by tweet type for each condition. As only 1 prompt was used per tweet for the *no identities* condition, the detection score is based only on the percentage of times a post was classified as misinformation across 30 runs. Within the context of this analysis, a higher percentage of detected misinformation for the misinformation tweets indicates a correct classification, while misinformation detection for the other tweet categories (eg, corrections and neutral) indicates a false positive. As seen in Table 2, when no identities were included in the prompt, ChatGPT correctly identified misinformation in 68.1% (SD 41.2%) of the tested posts on average. However, when all identities were included, the accuracy dropped to 29.3% (SD 31.6%) on average and was the lowest when only political identity was included (mean 19.2%, SD 29.2%).

For the other tweet types used to assess false positives, ChatGPT was less likely to classify a post as misinformation in the *all identities* condition when tweets contained guideline-unaligned (mean 3.8%, SD 5.7%) and guideline-aligned (mean 4.3%, SD 9.6%) sentiment compared with when no identities were included in the prompt (guideline unaligned: mean 8.9%, SD 11.3%; guideline aligned: mean 8.3%, SD 16.0%). False positives were rarely detected for sentiment tweets in the *only political identity* condition (guideline unaligned: mean 0%, SD 0%; guideline aligned: mean 1.1%, SD 3.0%). In the *all identities* condition, 10.8% (SD 16.6%) of the corrections were incorrectly classified as misinformation on average similar to the *no identities* condition (mean 13.3%, SD 17.3%). Corrections were least likely to be classified as misinformation in the *only political identity* condition (mean 2.5%, SD 4.5%). ChatGPT was also slightly more likely to classify neutral posts as misinformation (mean 3.9%, SD 6.2%) when all tested identities were included in the prompt, but it never classified neutral posts as containing misinformation in the *no identities* or *only political identity* conditions. Overall, the results show that false positives for nonmisinformation tweet types were typically less frequent in the *all identities* and *only political identity* conditions compared with the *no identities* condition.

**Table 2:** Average percentage detected as misinformation by tweet type^a^.

Tweet type	True or false positive?	All identities (%), mean (SD)	Only political identity (%), mean (SD)	No identities (%), mean (SD)
Misinformation	True	29.3 (31.6)	19.2 (29.2)	68.1 (41.2)
Unaligned sentiment	False	3.8 (5.7)	0 (0)	8.9 (11.3)
Aligned sentiment	False	4.3 (9.6)	1.1 (3)	8.3 (16)
Corrections	False	10.8 (16.6)	2.5 (4.5)	13.3 (17.3)
Neutral	False	3.9 (6.2)	0 (0)	0 (0)

^a^Higher percentage of detected misinformation reflects true positives (ie, correct classifications) for misinformation tweets. Scores for other tweet types (guideline-unaligned and guideline-aligned sentiment, corrections, and neutral) reflect false positives.

Table 3 shows the differences in misinformation detection accuracy by the specific identities tested in the prompts. In this table, the average percentage of correctly detected misinformation tweets are compared across identities. The results show that the type of political identity included in the prompt had little effect on accuracy, with conservative identities showing a 30.4% (SD 31.2%) accuracy on average compared with 28.1% (SD 32%) for liberal identities. The type of place and education tested also showed little difference in misinformation accuracy. The types of religious identity showed a bigger difference in accuracy: prompts including an atheistic identity had an accuracy of 33% (SD 32.8%) on average compared with a religious identity at 25.6% (SD 30%). Furthermore, prompts that included a narcissistic identity showed higher accuracy at classifying misinformation tweets compared with an empathetic identity (mean 32.1%, SD 30.1% vs mean 26.4%, SD 32.8%, respectively).

**Table 3:** Average percentage detected as misinformation by identity (misinformation tweets only)^a^.

Identity		Mean accuracy at classifying misinformation tweets (%), mean (SD)
**Political**		
	Conservative	30.4 (31.2)
	Liberal	28.1 (32)
**Religious**		
	Atheistic	33 (32.8)
	Religious	25.6 (30)
**Place**		
	Rural	29.5 (32.1)
	Urban	29 (31.1)
**Education**		
	Graduate	28.1 (31.6)
	High school	29.2 (31.4)
	Undergraduate degree	30.5 (31.9)
**Personality**		
	Empathetic	26.4 (32.8)
	Narcissistic	32.1 (30.1)

^a^As this table reports results on misinformation tweets only, all scores reflect true positives.

Table 4 compares differences in classification accuracy by each misinformation tweet. Accuracy scores are the percentage of times a post was classified as misinformation across the 30 runs for all tested prompts in each condition. Differences in accuracy percentages between the *all identities* and *no identities* conditions were also calculated. Misinformation tweets that are italicized signify posts that require contextual knowledge surrounding the discourse and the specific time period to correctly identify the false information they contain. The nonitalicized tweets signify posts where the language used reflects clear statements of misinformation and where “COVID” is explicitly mentioned.

**Table 4:** Comparing differences in misinformation classification accuracy between the all identities and no identities conditions by individual tweet^a^.

Tweet text	Mean accuracy: all identities (%), mean (SD)	Accuracy: no identities (control, N=30), n (%)	Difference (no identities–all identities; %)
“Friendly reminder the only reason DC Swamp Rats are against Hydroxychloroquine is because Big Pharma can’t make money off it It’s too cheap and easily accessible”	10.7 (8)	27 (90)	79.3
“Btw hydroxychloroquine cures Covid.”	28.3 (23)	28 (93)	65.1
“#HydrochloroquineWorks Early use of Hydroxychloroquine Azithromycin and Zinc as soon as symptoms appear reduces hospitalisations and fatalities by around 80%. But there’s no money in it for Big Pharma. And it destroys Bill Gates mass vaccination agenda. #Covid19”	37.6 (19.1)	29 (97)	59
“‘*Bacterial Pneumonia Caused Most Deaths in 1918 Influenza Pandemic’ - now we see why they want bacteria soaked masks on all of us....*”^b^	11.1 (12.7)	21 (70)	58.9
“COVID-19 syringes will have microchips on outside, not in vaccine. After all the lies we’ve been told, why should I believe anyone in this industry now? I smell something rotten.”	41.7 (27.3)	29 (97)	55
“I’m making a direct appeal to stop with the child abuse and take your kids out of the mask. The risk of kids dying of Covid is closer to 0 than kids dying of Pneumonia. Masks cause more harm than good! BAN SCHOOL MASK MANDATES”	46.2 (20.1)	30 (100)	53.8
“*Dr [redacted], who resigned from W.H.O because they tried to have swine-flu declared a pandemic, says that no vaccine can be safe without a minimum of 5-10 years development/testing. I trust him. No vaccine for me. Will use my immune system*”	16.3 (9.5)	20 (67)	50.4
“Can public health officials get any more stupid? Putting masks on children is idiotic. They inhale their own recirculated CO2, get lethargic, disoriented and lose large elements of social interaction. Masks don’t work anyway. Putting them on children is close to criminal.”	53.8 (27.7)	30 (100)	46.2
“*We’ve really gotten to a point where masks are more of a symbol of power than an attempt to control the pandemic. If you're powerless (kids in schools, service workers) you wear a mask b/c they can make you. If you’re powerful, you don’t b/c they can’t.*”	0.4 (1.6)	1 (3)	2.9
“*Big Pharma Whistleblower comes forward with documents proving ‘THERE IS NO VIRUS’ & no #vaccine is safe!*”	97.9 (3.2)	29 (97)	–1.3
“Consult your doctor before using Hydroxychloroquine. Side effects may include: profound understanding that COVID-19 is a treatable illness; sudden awareness of having been lied to; feelings of rational thought; an aversion to fear-based living; furious anger at China...”	1.7 (2.9)	0 (0)	–1.7
“*I am old enough to remember when ‘breakthrough cases‚’ were called ‘vaccine failures’.*”	5.3 (6.1)	1 (3)	–1.9

^a^Averages for *all identities* condition were generated by calculating the percentage of true positives across the 30 runs for each prompt request and then averaging the percentage of true positives across all 48 identity combinations. *No identities* condition shows the percentage of true positives across the 30 runs for each misinformation tweet.

^b^Italicized text indicates posts that are context dependent (ie, do not explicitly mention COVID-19).

The results show a high degree of variance in classification accuracy by each misinformation tweet; for example, the tweet “Big Pharma Whistleblower comes forward with documents proving ‘THERE IS NO VIRUS’ & no #vaccine is safe!” was correctly classified as misinformation >96% of the time on average for both the *all identities* and *no identities* conditions, while “Consult your doctor before using Hydroxychloroquine. Side effects may include: profound understanding that COVID-19 is a treatable illness; sudden awareness of having been lied to; feelings of rational thought; an aversion to fear-based living; furious anger at China...” was correctly classified less than 2% of the time on average, regardless of including identities. Furthermore, the accuracy of misinformation detection was greatly impacted by the addition of identities in the prompt. In the *no identities* condition, the tweets “Friendly reminder the only reason DC Swamp Rats are against Hydroxychloroquine is because Big Pharma can’t make money off it. It’s too cheap and easily accessible” and “Btw hydroxychloroquine cures Covid” were correctly classified as misinformation in 90% (27/30) and 93% (28/30) of the runs; however, the accuracy dropped to an average of 10.7% (SD 8%) and 28.3% (SD 23%), respectively, when all identities were included. Overall, more than half of the tested misinformation tweets (7/12, 58%) showed a decrease in accuracy of at least 50% when prompt identities were included. When comparing tweets containing explicit misinformation to those that were context dependent, there was no consistent pattern of differences in accuracy.

Table 5 compares differences in classification accuracy based on whether liberal or conservative identities were used in the prompt for the only political identity condition. A column for the accuracy scores of each tweet for the no identities condition has also been included for comparison. The results show that for most of the misinformation tweets (11/12, 92%), there was little difference in detection accuracy, regardless of assigned political identity. The exception was the tweet “Can public health officials get any more stupid? Putting masks on children is idiotic. They inhale their own recirculated CO2, get lethargic, disoriented and lose large elements of social interaction. Masks don’t work anyway. Putting them on children is close to criminal,” where ChatGPT was 60% more likely to classify it as misinformation when a liberal prompt was used compared with a conservative prompt. Notably, for more than half of the tested tweets (7/12, 58%), there was a difference in misinformation detection accuracy of at least 50% between the no identities and only political identity conditions; for example, in the no identities condition, the tweet “Btw hydroxychloroquine cures Covid” was correctly classified as misinformation 93% (28/30) of prompt runs but 0% for both liberal and conservative identities in the only political identity condition.

**Table 5:** Differences in misinformation classification accuracy between conservative and liberal identity prompts by individual tweet: only political identity condition (N=30 runs).

Tweet text	Accuracy: liberal identity, n (%)	Accuracy: conservative identity, n (%)	Difference (liberal identity–conservative identity; %)	Accuracy: no identities (control), n (%)
“Can public health officials get any more stupid? Putting masks on children is idiotic. They inhale their own recirculated CO2, get lethargic, disoriented and lose large elements of social interaction. Masks don’t work anyway. Putting them on children is close to criminal.”	21 (70)	3 (10)	60	30 (100)
“I’m making a direct appeal to stop with the child abuse and take your kids out of the mask. The risk of kids dying of Covid is closer to 0 than kids dying of Pneumonia. Masks cause more harm than good! BAN SCHOOL MASK MANDATES”	14 (47)	12 (40)	6.7	30^a^ (100)
“Friendly reminder the only reason DC Swamp Rats are against Hydroxychloroquine is because Big Pharma can’t make money off it It’s too cheap and easily accessible”	2 (7)	0 (0)	6.7	27^a^ (90)
“Btw hydroxychloroquine cures Covid.”	0 (0)	0 (0)	0	28^a^ (93)
“#HydrochloroquineWorks Early use of Hydroxychloroquine Azithromycin and Zinc as soon as symptoms appear reduces hospitalisations and fatalities by around 80%. But there’s no money in it for Big Pharma. And it destroys Bill Gates mass vaccination agenda. #Covid19”	13 (43)	13 (43)	0	29^a^ (97)
“‘*Bacterial Pneumonia Caused Most Deaths in 1918 Influenza Pandemic’ - now we see why they want bacteria soaked masks on all of us....*”^b^	0 (0)	0 (0)	0	21^a^ (70)
“*Dr [redacted], who resigned from W.H.O because they tried to have swine-flu declared a pandemic, says that no vaccine can be safe without a minimum of 5-10 years development/testing. I trust him. No vaccine for me. Will use my immune system*”	1 (3)	1 (3)	0	20^a^ (67)
“*We’ve really gotten to a point where masks are more of a symbol of power than an attempt to control the pandemic. If you’re powerless (kids in schools, service workers) you wear a mask b/c they can make you. If you’re powerful, you don’t b/c they can’t.”*	0 (0)	0 (0)	0	1 (3)
“Consult your doctor before using Hydroxychloroquine. Side effects may include: profound understanding that COVID-19 is a treatable illness; sudden awareness of having been lied to; feelings of rational thought; an aversion to fear-based living; furious anger at China...”	0 (0)	0 (0)	0	0 (0)
“*I am old enough to remember when ‘breakthrough cases‚’ were called ‘vaccine failures’*.”	0 (0)	0 (0)	0	1 (3)
“COVID-19 syringes will have microchips on outside, not in vaccine. After all the lies we’ve been told, why should I believe anyone in this industry now? I smell something rotten.”	2 (7)	10 (3)	–3.3	29^a^ (97)
“*Big Pharma Whistleblower comes forward with documents proving ‘THERE IS NO VIRUS’ & no #vaccine is safe!*”	25 (83)	93 (28)	–10	29 (97)

^a^Instances where there’s a difference of at least 50% in classification accuracy between the *no identities* condition and both conservative identity and liberal identity prompts.

^b^Italicized text indicates posts that are context dependent (ie, do not explicitly mention COVID-19).

### Identity Mentions

For each response, ChatGPT was asked to explain why it classified a post as either containing or not containing misinformation. Within the *all identities* condition (ie, political, religious, education, place, and personality), we calculated the percentage of times an identity was mentioned at least once in each response to assess whether ChatGPT weighs identities differently in importance when classifying misinformation. Table 6 shows the average percentage of times each identity is mentioned at least once across responses from the *all identities* condition. Political identities were mentioned the most often, with responses mentioning liberal identities 55.9% (SD 30.2%) times on average and conservative identities 66.8% (SD 32.9%) times. Religious identities were mentioned almost twice as often on average compared to atheistic identities (mean 46.6%, SD 28.7% vs mean 23.4%, SD 20.5%, respectively). For educational status, undergraduate degree was mentioned the least often (mean 30.6%, SD 31.1%) compared to high school (mean 58.7%, SD 34.7%) and graduate education (mean 51.5%, SD 37.2%). Place was mentioned the least often of the tested identities, with rural being mentioned slightly more often than urban (mean 25.1%, SD 29.3% vs mean 21.4%, SD 23.5%, respectively). When comparing personality traits, being empathetic was mentioned more often than being narcissistic (mean 34%, SD 20.7% vs mean 20.6%, SD 16.2%, respectively).

**Table 6:** Average percentage of identity mentions across all tweet types (n=1728 prompt requests)^a^.

Identity assignment		Mentions (%), mean (SD)
**Political**		
	Conservative	66.8 (32.9)
	Liberal	55.9 (30.2)
**Religious**		
	Atheistic	23.4 (20.5)
	Religious	46.6 (28.7)
**Education**		
	Graduate	51.5 (37.2)
	High school	58.7 (34.7)
	Undergraduate degree	30.6 (31.1)
**Place**		
	Rural	25.1 (29.3)
	Urban	21.4 (23.5)
**Personality**		
	Empathetic	34 (20.7)
	Narcissistic	20.6 (16.2)

^a^The percentage of identity mentions across the 30 runs for each prompt request was first calculated and then averaged across all 1728 prompt requests based on identity assignment.

Table 7 shows the average number of identity mentions across all responses broken out by tweet classification. Compared to the percentage of mentions across all tweet types, political identities were mentioned more often on average for tweets containing misinformation (68.6%, SD 30%) and guideline-aligned sentiment (71.4%, SD 27.4%). Religious identity was also more likely to be mentioned in misinformation tweets compared with all tweets (mean 40.9%, SD 29.4% vs mean 35%, SD 27.5%, respectively), while personality was mentioned more often for guideline-aligned sentiment tweets compared to all tweets (mean 35.3%, SD 22.2% vs mean 27.3%, SD 19.7%, respectively). Compared with all tweet types, responses to neutral tweets were more likely to mention education (mean 53.1%, SD 34.9% vs mean 46.9%, SD 36.4%) and place (mean 32.6%, SD 31.1% vs mean 23.2%, SD 26.6%) and less likely to mention political (mean 56.9%, SD 31.2% vs mean 61.4%, SD 32%), religious (mean 22.2%, SD 18.5% vs mean 35%, SD 27.5%), and personality (mean 19.6%, SD 15.2% vs mean 27.3%, SD 19.7%) identities.

**Table 7:** Average percentage of identity mentions by tweet classification (n=1728 prompt requests)^a^.

Tweet classification	Identity mentions in classification reason by tweet type (%), mean (SD)				
	Political	Religious	Education	Place	Personality
All types	61.4 (32)	35 (27.5)	46.9 (36.4)	23.2 (26.6)	27.3 (19.7)
Misinformation	68.6 (30)	40.9 (29.4)	39.7 (34.1)	19.9 (25)	29.5 (20)
Guideline-unaligned sentiment	55.9 (34.5)	32.4 (27)	47.5 (37.3)	19.9 (24.4)	24.4 (18.7)
Guideline-aligned sentiment	71.4 (27.4)	39.1 (27.1)	26.3 (28.4)	18.7 (21.6)	35.3 (22.2)
Corrections	46.9 (31.5)	34.4 (27.6)	75.3 (29.2)	28.5 (28.5)	25.3 (18.1)
Neutral	56.9 (31.2)	22.2 (18.5)	53.1 (34.9)	32.6 (31.1)	19.6 (15.2)

^a^The percentage of identity mentions across the 30 runs for each prompt request was first calculated and then averaged across all 1728 prompt requests based on tweet classification.

## Discussion

### Principal Findings

The findings reveal that asking ChatGPT to role-play social identities reduced its accuracy in classifying COVID-19–related misinformation. When we did not include identity cues in the prompts, ChatGPT correctly detected 68.1% (SD 41.2%) of the misinformation tweets when averaged across all tested runs. However, this accuracy decreased to 29.3% (SD 31.6%) on average in the condition where all identities were included and further declined to 19.2% (SD 29.2%) when testing only political identity, reflecting our expectation that adding identity cues would impact classification accuracy even when prompting ChatGPT with a specific definition of misinformation. ChatGPT’s misinformation detection accuracy in the *no identities* condition was similar to human performance when tasked to detect misinformation in the same tweets tested in this study: Kaufman et al [27] found that crowdsourced workers from Amazon Mechanical Turk correctly detected misinformation in 65.1% of the tweets on average [27]. However, ChatGPT’s performance was lower than that of undergraduate students, who correctly classified 77.7% of the misinformation tweets on average [27]. These comparisons with human performance suggest that specific groups of people may be able to outperform ChatGPT on misinformation detection.

ChatGPT was also able to distinguish sentiments that expressed opinions not aligned with public health guidelines from misinformation: guideline-unaligned sentiment tweets were incorrectly classified as misinformation in only 8.9% (SD 11.3%) of the runs on average in the *no identities* condition. Furthermore, correction tweets were more likely to have false positives than both guideline-aligned and guideline-unaligned sentiment tweets across all conditions. This may indicate that ChatGPT is more likely to label posts as nonfactual if they include declarative statements, as seen with corrections, compared with posts only expressing opinions.

While our findings show that the inclusion of identities decreases misinformation detection accuracy, it remains uncertain whether ChatGPT adopts views aligned with social identities. A closer examination reveals little variation in responses across the identity categories. When assigned a conservative identity, ChatGPT identifies misinformation with nearly the same frequency as it does when assigned a liberal identity, regardless of the tweet type. In the condition testing only political identity, there was only a single tweet that liberal prompts were 60% more likely to correctly classify as misinformation than conservative prompts. However, the majority of misinformation tweets (11/12, 92%) showed a difference of ≤10% between prompts in classification accuracy (7/12, 58% showed a difference of 0%). This is unexpected because conventional wisdom suggests that conservative identities would be more accepting of COVID-19–related misinformation, while liberal identities would be more skeptical. The uniformity in ChatGPT’s responses indicates a potential limitation in its ability to differentiate between polarized perspectives when asked to engage in role-play.

For many of the misinformation tweets (7/12, 58%), the inclusion of only political identity, regardless of being liberal or conservative, decreased detection accuracy by >50% compared with performance in the *no identities* condition. When investigating the reasons given for the classification decisions, we found ChatGPT’s justifications to be inconsistent. An example is shown in Textbox 2, which includes reasons for classifying the tweet “Btw hydroxychloroquine cures Covid,” where there was a difference of 93% in misinformation detection accuracy between the *no identities* and *only political identity* conditions. When no prompt identities were included, ChatGPT correctly classified the post as misinformation, stating that the claim that hydroxychloroquine cures COVID-19 “is false and not supported by scientific evidence.” However, when either liberal or conservative prompts were used, ChatGPT stated that the post did not contain misinformation because there was “no conclusive scientific evidence” and that the tweet presented “a viewpoint that is subject to ongoing scientific debate,” which is directly contradictory to the previous explanation.

**Examples of reasons provided by ChatGPT for misinformation classification.**

**Misinformation tweet**

- “Btw hydroxychloroquine cures Covid.”

**Reasons for *not* classifying tweet as misinformation**

- Liberal prompt (*only political identity* condition): “As a Liberal, I will evaluate the tweet based on the definition of misinformation provided. The tweet states that hydroxychloroquine cures Covid. However, it is important to note that the use of hydroxychloroquine as a cure for Covid-19 is a controversial topic. While some studies have shown potential benefits, there is not enough conclusive evidence to support the claim that hydroxychloroquine is a definitive cure for Covid-19. Therefore, this tweet does not contain misinformation as it presents a viewpoint that is subject to ongoing scientific debate and does not deliberately intend to deceive.”
- Conservative prompt (*only political identity* condition): “As a Conservative, I would base my judgment on scientific evidence and expert consensus. Currently, there is no conclusive scientific evidence to support the claim that hydroxychloroquine cures Covid. The tweet may be expressing a personal opinion or anecdotal evidence, but it does not meet the criteria of deliberate misinformation.”

**Reason for classifying tweet as misinformation**

- No *identities* condition (control): “The tweet contains misinformation because it claims that hydroxychloroquine cures Covid, which is false and not supported by scientific evidence.”

Our study also demonstrates that misinformation detection accuracy can vary widely by individual tweet. Of the 12 misinformation tweets, 8 (67%) showed a decrease of >40% in misinformation detection accuracy when all identities were included in the prompts compared with the *no identities* condition. However, there were no consistent differences in accuracy based on whether misinformation was explicit or context dependent. In general, some context-dependent misinformation tweets showed a decrease of >50% in accuracy when prompt identities were included, while others showed little difference in performance between the conditions. These inconsistencies may be a reflection of ChatGPT’s pretrained data set during experimentation because algorithms can improve at detecting implicit meanings in text when given domain-specific data.

The identities mentioned in ChatGPT’s explanations for each classification decision varied in frequency, which may reflect that ChatGPT weighs the importance of identities differently; for instance, political identity was referenced in 61.4% (SD 32%) of responses on average compared with locality (23.2%, SD 26.6%), suggesting a greater emphasis on stated political beliefs over locality when assessing misinformation. While this pattern suggests that ChatGPT may be attributing varying levels of importance to different identities in determining the credibility of health-related information, the “black box” nature of LLMs [49,50] makes it impossible to determine definitively that the output given in the classification explanations corresponds to how factors are actually weighted in ChatGPT’s evaluation process. Further research and experimentation are needed to investigate how ChatGPT and other LLMs, such as Google Gemini, weigh cues mentioned in prompts when generating responses.

As demonstrated in this study, ChatGPT correctly classified misinformation in 68.1% (SD 41.2%) of the tested posts on average when no identity cues were included in the prompt. While these results are promising, completely relying on ChatGPT to identify misinformation without oversight from human coders may be premature based on current versions of LLMs. In the case of novel events where training data sets do not correspond to emerging circumstances, researchers in infodemiology and related fields should consider hybrid approaches for content coding that incorporate both human annotators and AI techniques (refer to the study by Haupt et al [16] for an example). Human annotators may also be more adept at detecting implicit meanings in text, especially in crises where scientific evidence and circumstances are frequently shifting. However, it is worth noting that a lack of contextual knowledge can be a concern among humans as well, as seen in previous work showing that human performance in sarcasm detection was similarly low compared with machine learning approaches [51].

### Implications of Using ChatGPT in Infodemiology

The use of role-play in ChatGPT prompts has significant implications for health communication professionals. In addition to detecting misinformation in social media posts, this functionality can be used to assist in tailoring messaging for targeted groups based on demographic and psychological factors. More specifically, users can ask LLMs to generate message options using role-play prompts and then further edit the messages before testing responses from humans. This functionality complements recent efforts that develop “personas” or “cognitive phenotypes” to produce more nuanced depictions of public response toward health issues [30,52-55]. In practice, personas can be developed to characterize different types of reactions, perceptions, beliefs, and narratives that people may have toward future health crises while accounting for personality traits, situational circumstances, and demographic factors. LLMs can then be used to generate options for tailored messages, recommendations, or interventions for each persona that can be deployed in targeted health promotion or communication activities (eg, debunking misinformation).

It is important to note that while the ability to generate customized language that resonates with particular groups can greatly extend the reach and impact of public health campaigns, this functionality presents potential risks because it can also be adopted by actors with malintent to craft more effective conspiracy messaging and false narratives. As output from LLMs is becoming increasingly indistinguishable from human responses [56], chatbots using LLMs raise particular concerns because they can be used to create fake accounts that deceive users by mimicking the language patterns of targeted identities (refer to the studies by De Angelis et al [11], Park et al [57], and Hajli et al [58] for detailed discussions on the risks posed by chatbots and AI systems for manipulative tactics, such as fraud and disinformation campaigns, and the study by Arnold et al [59] for a more general review of using chatbots to address public health concerns).

Our findings suggest that, when classifying misinformation, ChatGPT may place different levels of importance on identities when assigned multiple roles. While we are unable to make a definitive conclusion concerning ChatGPT’s use of identity weighting, these findings still raise the question of whether responses from LLMs *should* weigh social identities differently when included in prompts, and if so, how the weights should be distributed. In cases where a group based on either demographic factors or psychological dispositions is particularly vulnerable to specific types of misinformation or narratives, should LLMs account for this difference in susceptibility when generating responses? Furthermore, how should changes in language use and definitions of identities over time be accounted for? At the present moment, this discourse is mostly speculative and requires further discussion among researchers, officials, and health practitioners to consider the ethical implications of using AI technologies.

Another factor to consider in the use of LLMs by the general public is potential functionalities that ingest metadata from users (eg, cookie files, profile data, and search histories). When a request is submitted to an LLM, it could construct identity profiles using these metadata, which can then subsequently alter its response even if the identity is not explicitly mentioned in the prompt. In other words, this functionality would result in users receiving tailored responses regardless of whether it was formally requested. A similar phenomenon is observed in newsfeed algorithms across social media platforms and search engine results, where the information presented to users is typically customized based on self-reported profile information and previous online behaviors [60,61]. Responses from LLMs that are tailored to identities could potentially exacerbate political polarization and echo chambers that are already prominent in online spaces.

### Limitations

There are limitations that should be considered for this study. As ChatGPT is based on a corpus of English-language data from predominantly Western sources, its responses are not likely to represent perspectives from other countries and languages where fewer data are available. This study also focuses on COVID-19–related misinformation within the context of US-centered discourse and tested prompts with identities that may only be relevant within the United States. Further work is needed to assess ChatGPT’s ability to detect misinformation for other topics and cultural contexts. Another limitation is that identity was only tested using the role-play option in the prompts. It is likely that explicitly stating values, beliefs, and behaviors associated with identities may influence output as opposed to only mentioning the identity in the prompt without further context.

### Conclusions

Our findings show that ChatGPT’s performance when classifying misinformation is greatly influenced when social identities are included in the prompts, as evidenced by the stark contrast in accuracy between the *all identities* and *no identities* conditions. However, the degree of influence remains uncertain, as indicated by the minimal differences observed between categories within the same identity. Furthermore, ChatGPT’s use of its assigned identities is inconsistent: it places considerable emphasis on certain identities in its reasoning explanations, such as political beliefs, while downplaying others, such as locality. As the use of LLMs by researchers, health officials, and the general public will likely continue to grow in upcoming years, these considerations will need to be addressed to ensure effective use of this powerful tool while mitigating potential consequences, particularly in the context of future health emergencies and infodemics.

## Abbreviations

AI: artificial intelligence
LLM: large language model

## Data Availability

The data sets generated and analyzed during this study as well as the ChatGPT-generated responses are available in the GitHub repository [62]. The repository also includes our code and detailed instructions on how to reproduce the study experiments.
